# Spine morphogenesis and synapse formation in tubular sclerosis complex models

**DOI:** 10.3389/fnmol.2022.1019343

**Published:** 2022-12-20

**Authors:** Tadayuki Shimada, Kanato Yamagata

**Affiliations:** ^1^Child Brain Project, Tokyo Metropolitan Institute of Medical Science, Tokyo, Japan; ^2^Department of Psychiatry, Takada Nishishiro Hospital, Niigata, Japan

**Keywords:** tuberous sclerosis complex, spine morphology, synapse formation, rapamycin, syntenin

## Abstract

Tuberous sclerosis complex (TSC) is caused by mutations in the *Tsc1* or *Tsc2* genes, whose products form a complex and inactivate the small G-protein Rheb1. The activation of Rheb1 may cause refractory epilepsy, intellectual disability, and autism, which are the major neuropsychiatric manifestations of TSC. Abnormalities in dendritic spines and altered synaptic structure are hallmarks of epilepsy, intellectual disability, and autism. In addition, spine dysmorphology and aberrant synapse formation are observed in TSC animal models. Therefore, it is important to investigate the molecular mechanism underlying the regulation of spine morphology and synapse formation in neurons to identify therapeutic targets for TSC. In this review, we focus on the representative proteins regulated by Rheb1 activity, mTORC1 and syntenin, which are pivotal downstream factors of Rheb1 in the alteration of spine formation and synapse function in TSC neurons.

## 1 Introduction

Tuberous sclerosis complex (TSC) is a genetically inherited disorder. The estimated incidence of TSC at birth is 1:6,000-10,000. TSC is characterized by the formation of a benign tumor, called a hamartoma, in a wide range of tissues, including the brain, eyes, skin, kidneys, heart, and lungs, resulting in a variety of symptoms and complications (Gómez, 1995). Hamartoma in the brain correlates with the onset of refractory epilepsy (Cusmai et al., 1990; Weiner et al., 2006), intellectual disability (Goodman et al., 1997), and autism spectrum disorders (ASDs) (Doherty et al., 2005; Numis et al., 2011). Heterozygous mutation in either the *Tsc1* or *Tsc2* gene induces these phenotypes; therefore, these two genes are responsible for TSC (Consortium, 1993), and TSC is an autosomal-dominant disorder.

The *Tsc1* gene encodes hamartin. Mutations in *Tsc1* are associated with an usually milder clinical phenotype in patients (Cheadle et al., 2000; Henske et al., 2016); on the other hand, *Tsc2* mutation accounts for the majority of cases (∼70%) and is associated with greater disease severity, such as earlier seizure onset, more cases of subependymal nodules, and a lower cognition index than *Tsc1* mutation (Cheadle et al., 2000; Henske et al., 2016). The Hamartin protein contains a Tsc2 protein-binding coiled-coil domain, and the Tsc2 protein, called tuberin, includes a hamartin interaction domain and a GTPase-activating domain (Henske et al., 2016; Peron et al., 2018). The interaction between hamartin/Tsc1 protein and tuberin/Tsc2 protein may stabilize the protein complex (Benvenuto et al., 2000), and GTPase-activating activity is the fundamental function of the Tsc1/Tsc2 complex. The loss of its function causes severe defects in downstream signaling, resulting in severe phenotypes.

Dendritic spines are the main sites of excitatory synapse formation and exhibit a wide variety of shapes. Altered dendritic spines can be observed in neurodevelopmental and psychiatric disorders. The pathological alteration in spines can be classified into two categories: abnormalities in spine density and abnormalities in spine morphology. Pathologies of the density include remarkable increases and decreases in spine density, and pathologies of morphology include changes in spine size and distorted spine shape. Human postmortem and model animal studies have reported that a change in spine density and aberrant thin spine shape were observed in the neurons of genetic disorders associated with intellectual disability, ASD, and refractory epilepsy, such as Fragile-X syndrome (Irwin et al., 2001; Bagni and Greenough, 2005), Angelman syndrome (Dindot et al., 2008; Kim et al., 2016), and Rett syndrome (Zhou et al., 2006; Xu et al., 2014). In addition, a pharmacological animal model of ASD and epilepsy exhibited abnormal spine density and morphology (Wong and Guo, 2013; Takuma et al., 2014). Pathological changes in spines may result in abnormal synaptic plasticity and lead to the symptoms of these disorders; however, signs of abnormal synaptic plasticity in these diseases are highly variable, and often opposite trends of spine alteration are observed. Although the type of abnormalities in dendritic spines greatly differs in various disorders, changes in spine number or morphology may be the original insult that initiates symptoms, which are also observed in TSC. Understanding the mechanism underlying spine alteration may allow the identification of new foci for therapeutic strategies. Here, we review the properties of Tsc1 and Tsc2 proteins in regulating dendritic spine morphogenesis and synapse formation by summarizing the signaling pathways that are altered in TSC.

## 2 Signaling pathways of the Tsc1/2 complex, proteins regulating Tsc1/2 and proteins regulated by Tsc1/2

**FIGURE 1:**
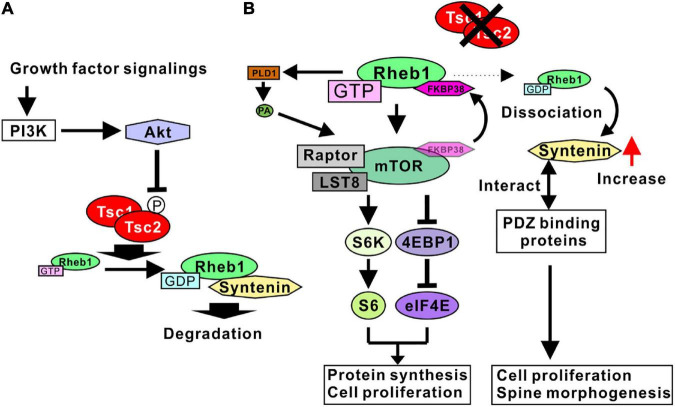
Signaling pathways regulated by Tsc1/2. **(A)** Growth factor signaling activates Akt through PI3K. Active Akt phosphorylates Tsc2, leading to the inhibition of TSC1/2 GAP activity. Tsc1/2 act as GAP proteins of Rheb1. The GDP-bound form Rheb1 binds to syntenin and is degraded by proteosomes. **(B)** Tsc1/2 loss of function results in an increase in GTP-form Rheb1 (active Rheb1). Active Rheb1 induces the activation of the mTORC1 complex by sequestering FKBP38 and PLD1-mediated PA production. Active mTORC1 promotes protein synthesis and cell proliferation (left). Reduction of GDP-form Rheb1 resulted in a dissociation of syntenin from Rheb1 and an increase in free syntenin. Syntenin interacts with various types of PDZ domain-binding proteins, leading to cell proliferation and spine malformation (right).

As mentioned above, the Tsc1/2 complex acts as a GTPase-activating protein (GAP). The target protein of Tsc1/2 GAP activity is a small G-protein Rheb1 (Inoki et al., 2003), and normal Tsc1/2 increases the GDP-bound form of Rheb1 (Figure 1A). A loss of function of Tsc1 or Tsc2 results in the activation of Rheb1 (Pan et al., 2004). Rheb1 was originally isolated as a protein upregulated by neuronal activity and named as the Ras homolog enriched in the brain (Yamagata et al., 1994). Further analyses have shown that Rheb1 protein is ubiquitously expressed in a variety of tissues, despite its name. Before Rheb1 was isolated as a target protein of Tsc1/2, it was shown that the Tsc1/2 protein inhibited the phosphorylation of ribosomal S6 kinase (S6K) and eukaryotic initiation factor 4E-binding protein (4EBP1) (Goncharova et al., 2002; Inoki et al., 2002), suggesting that Tsc1/2 mutation induces mammalian target of rapamycin complex 1 (mTORC1) activation through the activation of Rheb1 (Gao et al., 2002; Tee et al., 2002). As expected, mTORC1 is now accepted as a downstream target of Rheb1, and the GTP-bound form of Rheb1 can activate mTORC1 (Figure 1B). Because active mTORC1 causes cell proliferation and cell growth, it is consistent that Tsc1/2 mutation results in the formation of benign tumors in various organs.

Although active Rheb1 binds mTORC1 directly in living cells (Heard et al., 2014), the mechanism whereby Rheb1 activates mTORC1 has not been fully determined. Possible mechanisms underlying the Rheb1-mediated activation of mTORC1 have been demonstrated in several studies. One of the candidate mechanisms is the Rheb1-mediated inhibition of FK506 binding protein 38 (FKBP38), an endogenous mTOR inhibitor. Rheb1 directly interacts with FKBP38 in a GTP-dependent manner. The Rheb1-FKBP38 interaction sequesters FKBP38 and prevents the association between FKBP38 and mTOR, activating mTORC1 (Bai et al., 2007). Another candidate mechanism is phospholipase D 1 (PLD1)-mediated mTORC1 activation. PLD1 interacts with Rheb1, and the interaction increases the activity of PLD1 to produce phosphatidic acid (PA) (Heard et al., 2014). Because PA stabilizes mTOR and activates mTORC1, Rheb1-mediated PLD1 activation accounts for the mechanism of mTORC1 activation by Rheb1 (Figure 1B).

Recent studies have indicated that syntenin is another target protein regulated by Rheb1 activity. Syntenin protein can preferably interact with the GDP-bound form of Rheb1 (Sugiura et al., 2015). The Rheb1-syntenin complex is rapidly degraded by the proteasome. With normal Tsc1/2 activity, sufficient GTPase activity of Rheb1 supports the syntenin-mediated degradation of Rheb1, contributing to the downregulation of Rheb1 activity by decreasing the amount of Rheb1 protein. In addition, when Rheb1 is in a GTP-bound form, syntenin is easily dissociated from Rheb1 and is unlikely to be degraded by the proteasome. Therefore, the mutation of Tsc1/2 promotes an increase in syntenin protein levels through the dissociation of syntenin and active Rheb1 (Figure 1B; Sugiura et al., 2015). Syntenin is an adaptor protein containing two PDZ domains and has been shown to interact with a wide variety of proteins (Beekman and Coffer, 2008; Shimada et al., 2019). In addition, a loss of function of syntenin resulted in the impairment of cell division and cell growth in a variety of cancer cell lines (Kashyap et al., 2015), indicating that an increase in syntenin promotes cell division and proliferation. These results are consistent with hamartoma formation by Tsc1/2 mutation. Taken together, these results indicated that the Tsc1/2 complex regulates the cell proliferation signaling pathway governed by mTORC1 and syntenin through the regulation of Rheb1 activity (Figure 1B).

As Tsc1/2 regulates cell proliferation and cell growth, it is plausible that the activity of Tsc1/2 is regulated by growth factor signaling. One of the representative factors that is activated by growth factors, the PI3K (phosphatidylinositol-3 kinase)/Akt pathway, is involved in Tsc1/2 regulatory signaling. Tsc2 protein is phosphorylated by Akt (Inoki et al., 2002; Manning et al., 2002), and phosphorylation impairs the GAP activity of the Tsc1/2 complex (Huang and Manning, 2008). In addition, the overexpression of Tsc2 harboring alanine-substitution mutations at putative phosphorylation sites by Akt can predominantly block Akt-mediated activation of mTORC1 (Inoki et al., 2002; Cai et al., 2006). These results indicate that Tsc2 phosphorylation by Akt causes a loss of GAP activity and an increase in GTP-bound form of Rheb1 (Figure 1A), leading to mTORC1 activation and the induction of cell proliferation and cell growth.

Taken together, these results indicate that PI3K/Akt signaling negatively regulates Tsc1/2 by phosphorylation, and the Tsc1/2 complex inactivates Rheb1 protein. A loss of function of Tsc1/2 causes mTORC1 activation and an increase in syntenin through Rheb1 activation, promoting the cell proliferation observed in hamartoma cells with TSC pathology.

## 3 mTORC1-mediated regulation of spines and synapses in TSC neurons

It is well known that mTORC1 is a pivotal protein complex in the process of protein synthesis and cell proliferation (Hara et al., 1998; Schmelzle and Hall, 2000; Battaglioni et al., 2022). In brief, S6K and 4EBP1 are phosphorylated by mTORC1. Phosphorylated S6K induces the transcription of rDNA and promotes ribosome biosynthesis. Phosphorylation of 4EBP1 releases its binding from eukaryotic translation initiation factor 4E (eIF4E), enabling incorporation of eIF4E into the transcription initiation complex to start cap-dependent translation. Protein synthesis is highly involved in the regulation of synaptic efficacy, such as long-term potentiation (LTP). In particular, the long-lasting late phase of synaptic alteration is a protein synthesis-dependent event (Bramham and Wells, 2007; Bramham, 2008). Therefore, the regulation of mTORC1 is believed to be a key regulator of spine and synapse function.

Several studies have investigated the effect of mTORC1 activity on spine density and morphology in TSC models. The regulation of mTORC1 activity is believed to alter spine morphogenesis, but controversial results have been shown. Activating mTORC1 through the expression of constitutively active Akt (probably through Tsc1/2 inactivation) elongated the dendritic spines and decreased the density of spines, and inhibiting mTORC1 by rapamycin further reduced the spine density in cultured hippocampal neurons (Kumar et al., 2005). Other research indicated that activating mTORC1 by *Tsc1* knockout resulted in long and thick dendritic spines and decreased the density of spines; however, pharmacological inhibition of mTORC1 by rapamycin treatment led to long and thin spines in mouse hippocampal organotypic slice culture (Tavazoie et al., 2005). In addition, rapamycin treatment of *Tsc2* knockdown neurons further elongated the spines (Tavazoie et al., 2005). Yasuda et al. showed that a *Tsc2* heterozygous mutation (*Tsc2*^±^) reduced the spine width and increased the length of dendritic spines in cultured hippocampal neurons (Yasuda et al., 2014). Rapamycin treatment further elongated the spines in *Tsc2*^±^ neurons, but surprisingly, rapamycin did not change the spine morphology in control wild-type (WT) neurons. The authors also showed that the knockdown of *mTOR* further elongated the *Tsc2*^±^ dendritic spines and slightly altered WT spines (Yasuda et al., 2014). However, one paper showed different results regarding the effect of Tsc2 loss. Nie et al. (2015) indicated that the knockdown of *Tsc2* by short hairpin RNA (shRNA) resulted in shorter dendritic spines and reduced spine density in cultured hippocampal neurons. Rapamycin treatment normalized the spine length but did not change the spine density. In this case, overnight treatment with rapamycin was effective in normalizing spine morphology (Nie et al., 2015), whereas prolonged rapamycin treatment (6 to 9 days) was used in other papers (Kumar et al., 2005; Tavazoie et al., 2005; Yasuda et al., 2014). Only the short duration of rapamycin treatment may be helpful for normalizing mTORC1-mediated spine dysmorphogenesis.

**FIGURE 2:**
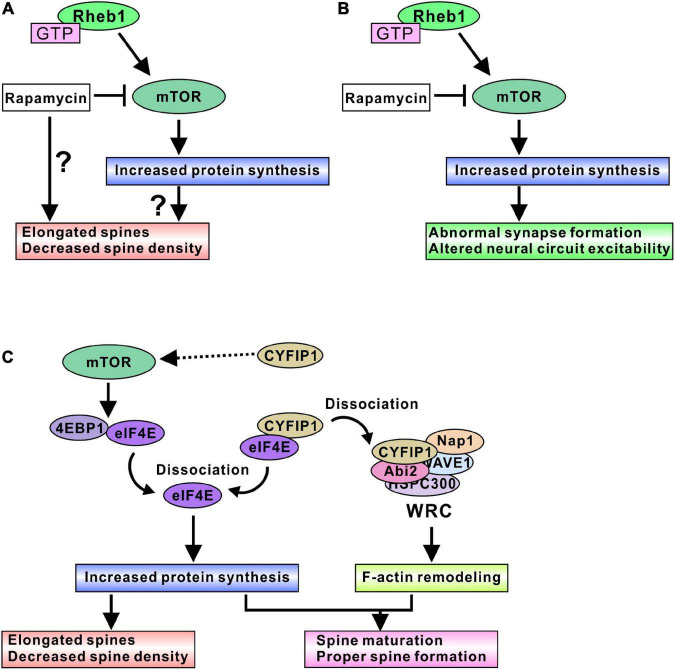
mTORC1-mediated spine and synapse regulation in TSC models. **(A)** Active mTORC1 may elongate dendritic spines and decrease spine density, probably through the increased protein synthesis. In addition, rapamycin treatment could aggravate the spine dysmorphogenesis. **(B)** Active mTORC1 alters synapse function and neural circuit excitability. Inhibition of mTORC1 by rapamycin and its derivative normalizes abnormal neural excitability, indicating mTORC1-mediated protein synthesis is involved in adequate synapse formation and function. **(C)** Schematic model of protein synthesis and spine morphogenesis. Active mTORC1 dissociates eIF4E from 4EBP1, and dissociated eIF4E promotes protein synthesis. Dysregulation of protein synthesis may cause elongated spine formation. However, rapamycin experiments suggest that modulation of protein synthesis alone does not provide the correct control of spine morphogenesis. CYFIP1 is another binding partner of eIF4E. Dissociation of eIF4E from CYFIP1 also promotes protein synthesis. Furthermore, dissociated CYFIP1 is incorporated into WRC protein complex and regulates F-actin remodeling, suggesting that CYFIP1-mediated protein synthesis control is associated with the F-actin remodeling changes. Therefore, regulation of both protein synthesis and F-actin remodeling could be required for the normal spine formation and maturation. Because excess amount of CYFIP1 activate mTORC1 signaling, CYFIP1 may be another regulator of mTORC1 activity.

Taken together, these results, although controversial, show various ways that the loss of function of the Tsc1/2 complex causes elongated dendritic spine formation and decreased spine density. Rapamycin treatment did not normalize the altered spine morphogenesis, further elongated the spines, and reduced the spine density (Figure 2A). Therefore, the loss of function of Tsc1/2 surely alters dendritic spine morphology; however, it is difficult to say that mTORC1 is a main regulatory protein that alters spine formation in TSC neurons. Research on fragile X syndrome model mice could explain the reason mTORC1 is not a pivotal regulator of spine morphogenesis. Fragile X mental retardation protein (FMRP) and cytoplasmic FMRP interacting protein 1 (CYFIP1) are involved in dendritic spine maturation, and the function of FMRP and the CYFIP complex can explain why rapamycin treatment did not normalize spine dysmorphogenesis. CYFIP1 and FMRP form a complex and interact with eIF4E protein (Napoli et al., 2008). The interaction of these proteins results in an inhibition of protein translation (Napoli et al., 2008). Synapse activation, such as brain-derived neurotrophic factor (BDNF) stimulation, induces a dissociation of CYFIP1 from eIF4E to promote protein synthesis (Napoli et al., 2008), and dissociated CYFIP1 is incorporated into a protein complex named the WAVE regulatory complex (WRC) to facilitate F-actin remodeling (De Rubeis et al., 2013). The interaction between CYFIP1 and WRC induces the mature form of spine morphogenesis (De Rubeis et al., 2013; Figure 2C). Knockdown of CYFIP1 impaired spine maturation and resulted in elongated spines in cultured neuron dendrites (De Rubeis et al., 2013). Interestingly, overexpression of CYFIP1 induced the activation of mTORC1 and increased the number of mature dendritic spines (Oguro-Ando et al., 2015) and rapamycin treatment normalized the “overmaturation” of dendritic spines (Oguro-Ando et al., 2015). These results suggest that spine maturation requires both adequate F-actin remodeling and sufficient protein synthesis (Figure 2C). CYFIP1 release from eIF4E can fulfill both conditions, whereas mTORC1-mediated 4EBP1 release from eIF4E regulates only protein synthesis. Rapamycin treatment could fail to cure the abnormal spine morphology.

Synaptic functions of mTORC1 are also well studied in TSC model neurons. In cultured mouse hippocampal neurons, *Tsc1* knockout induced high frequency and spontaneous network activity, and rapamycin treatment ameliorated the elevated spike frequency (Figure 2B), indicating that hyperactivity in TSC neurons is an mTORC1-dependent event (Bateup et al., 2013). An acute slice study using excitatory neuron-specific *Tsc1* knockout mice showed reduced intrinsic excitability but normal synaptically-driven excitability, and decreased inhibitory synaptic currents in *Tsc1* knockout mouse neurons. As a result, an excitatory/inhibitory synaptic imbalance was observed in *Tsc1* knockout neurons, and rapamycin treatment normalized the imbalance in mouse brain slices (Bateup et al., 2013). Neurons differentiated from TSC patient-derived induced pluripotent stem cells (iPSCs) exhibited altered neuronal excitability. Both *Tsc1*- and *Tsc2*-deficient iPSC-derived neurons showed increased firing rates compared with *Tsc1*^+/+^ and *Tsc2*^+/+^ neurons (Nadadhur et al., 2019; Winden et al., 2019; Alsaqati et al., 2020). Different iPSC cell line-derived neurons showed similar results, indicating that a higher rate of neuronal firing is the representative phenotype of the cultured TSC neurons. In addition, some studies showed highly synchronized bursts in TSC neurons (Nadadhur et al., 2019; Winden et al., 2019), whereas one study showed that TSC neurons exhibited lower synchronization of neuronal active bursts (Alsaqati et al., 2020). Rapamycin treatment normalized the higher firing rate and promoted greater synchrony of the active burst (Nadadhur et al., 2019; Winden et al., 2019). On the other hand, in one study, rapamycin treatment did not have any effect on neuronal network behavior in TSC neurons, but mTORC1 inactivation through pharmacological activation of Unc-51-like autophagy activating kinase 1 (ULK1) led to the recovery of neuronal synchronization (Alsaqati et al., 2020). The differences in iPSC cell lines and differentiation protocols may be due to the variety of phenotypes in active burst synchrony and rapamycin sensitivity (Winden et al., 2019; Alsaqati et al., 2020), or a relatively high concentration of rapamycin is required for normalization of neuronal activities. In addition, RT–qPCR analysis showed abnormal gene expression of excitatory/inhibitory synapse formation and function in TSC neurons. For example, glutamic acid decarboxylases (GAD), postsynaptic gamma-amino butyric acid (GABA) receptor subunits, the glutamate receptor (GluR) genes, and the presynaptic vesicular glutamate transporter (vGlut) were upregulated in TSC neurons, indicating that excitatory/inhibitory synapse function is dysregulated (Alsaqati et al., 2020). Furthermore, CD44 and sushi repeat protein X-linked 2 (SRPX2) showed increased expression (Winden et al., 2019). Because these gene products are involved in the promotion of synapse formation (Sia et al., 2013; Roszkowska et al., 2016), it is suggested that loss of Tsc2 function could promote synaptogenesis and induce hyperexcitability. Another study using human iPSC-derived neurons suggested that an increase in ion channel protein leads to neuronal excitability and synchrony. CRISPR/Cas9-mediated homogenous *Tsc2* knockout in neurons (*Tsc2*^–/–^) resulted in highly synchronized Ca^2+^ spikes and rapamycin treatment changed Ca^2+^ dynamics from synchronous to sporadic patterns (Hisatsune et al., 2021). The altered Ca^2+^ dynamics in *Tsc2* knockout neurons are caused by an increase in one of the L-type voltage-gated calcium channel proteins, Cav 1.3, and the increase in Cav 1.3 is an active-Rheb1-dependent event in iPSC-derived neurons, indicating that mTORC1 is involved in changes in Ca^2+^ dynamics in TSC neurons (Hisatsune et al., 2021).

A further additional clue explaining how mTORC1 regulates synaptic function *in vivo* was found in a study in rats. A recent study revealed that synapse input induced an accumulation of eIF4E in the spine head. After the tone-shock fear conditioning test, accumulation of eIF4E protein was observed in the spine heads of the rat brain (Gindina et al., 2021). There were more eIF4E-accumulated spines in the brains of trained rats at both the smaller (0.05–0.1 μm^2^) and larger (> 0.2 μm^2^) spine head areas of the range (Gindina et al., 2021). Other translation initiation factors (eIF4G1 and eIF2α) were also observed in spine heads of trained rat brains, suggesting that protein translation initiation can occur in dendritic spines after synapse inputs. Because eIF4E protein levels were not altered in TSC patient-derived neural progenitor cells (Martin et al., 2020), mTORC1 activation might increase free eIF4E. Therefore, synapse inputs may induce the translocation of active-mTORC1-dependent free eIF4E into spine heads and increase protein synthesis there, leading to a change in synapse excitability. In summary, mTORC1 activation seems to be involved in the alteration of synapse function in TSC neurons, probably through the increase in the synthesis of synaptic proteins, such as transporters, receptors of neurotransmitters, and ion channels. Rapamycin treatment normalized the impaired synapse functions by balancing the amount of synapse proteins (Figure 2B).

## 4 Syntenin-mediated regulation of spines and synapses in TSC neurons

Syntenin was originally isolated as a binding protein of syndecan-2, a postsynaptic protein (Grootjans et al., 1997). Because syntenin contains two PDZ domains and PDZ domains bind to the C-terminus of the partner protein, syntenin works as an adaptor protein. Subsequent studies have shown that syntenin has a variety of interacting partner proteins (Beekman and Coffer, 2008; Shimada et al., 2019). In its role as a multifunctional adaptor protein, syntenin has been implicated in neural cellular events, including developmental regulation of neuronal membrane architecture (Hirbec et al., 2005; Ro et al., 2010), synapse formation (Sugiura et al., 2015), and axonal elongation (Tomoda et al., 2004).

**FIGURE 3:**
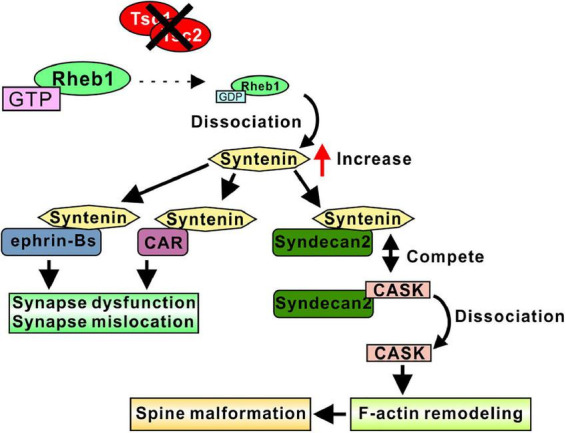
Syntenin-mediated spine and synapse regulation in TSC models. Loss of function of Tsc1/2 results in an increase in syntenin. Increased syntenin interacts with ephrin-B proteins, altering synapse function and synapse location. Syntenin binds to the CAR protein and regulates the mGluR compartment in the postsynaptic membrane, which may control synapse function. Syntenin competes with CASK for binding to Syndecan-2, indicating that increased syntenin dissociate CASK from Syndecan-2. Free CASK associate with F-actin and promote F-actin remodeling, resulted in a spine malformation. Therefore, the syntenin-Syndecan-2 interaction leads to elongated dendritic spines.

Because syntenin is degraded by interacting with GDP-form Rheb1, Rheb1 activation through the loss of function of Tsc1/2 resulted in an increase in syntenin (Sugiura et al., 2015). Consistent with the elongated dendritic spines in cultured *Tsc2*^±^ neurons, the overexpression of syntenin in cultured neurons resulted in the formation of longer spines, whereas *syntenin* knockdown and *syntenin* knockout in *Tsc2*^±^ neurons normalized the alterations in spine morphology. In addition, the overexpression of the Rheb1 mutant, which preferably has a GDP-bound form and strongly interacts with syntenin, also restored the abnormal spine formation in *Tsc2*^±^ neurons (Sugiura et al., 2015). Interestingly, syntenin-mediated elongated spines were normalized by the overexpression of calcium/calmodulin-dependent serine protein kinase (CASK), another syndecan-2 interacting protein. This result indicates that syntenin competes with CASK for syndecan-2. In another experiment, *syntenin* knockdown in WT neurons also resulted in the formation of elongated spines, suggesting that the balance of the protein amount between syntenin and CASK is important for normal spine morphogenesis (Sugiura et al., 2015). As CASK is known to associate with F-actin via its binding protein to protein 4.1 to promote F-actin remodeling (Biederer and Sudhof, 2001) and may mediate spine morphogenesis through syndecan-2, an increase in syntenin could dissociate CASK from syndecan-2, resulting in elongated spine formation (Figure 3). Furthermore, *CASK* is the gene responsible for Ohtahara syndrome, one of the most severe and earliest forms of epilepsy (Saitsu et al., 2012), and mutation in CASK resulted in a synapse dysfunction and induced epileptic episodes (Hsueh, 2009; Giacomini et al., 2021). The findings from another study indicated that the interaction between syntenin and syndecan-2 is necessary for the return of syndecan-2 to the plasma membrane from intracellular membrane structures (Zimmermann et al., 2005), and the overexpression of mutant syndecan-2, which cannot interact with syntenin, induces elongated spines (Ethell and Yamaguchi, 1999), indicating that postsynaptic localization of syndecan-2 via syntenin is important for the maturation of dendritic spines.

Syntenin regulates synapse formation by interacting with ephrin-B family proteins. The overexpression of syntenin and ephrin-B3 increased the number of spines and synapses formed on spines (Xu et al., 2011). The deletion of the syntenin PDZ domain abolished these changes, indicating that the interaction between ephrin-B3 and syntenin is required for spine formation. The knockdown of ephrin-B3 in *Tsc2*^±^ neurons normalized spine shape and synapse formation (Sugiura et al., 2015). In addition, the overexpression of syntenin and ephrin-B1 or ephrin-B2 also increased the number of synapses (McClelland et al., 2009). This synapse number regulation is dependent on ephrin-B/Eph-B signal transduction. The interaction between syntenin and ephrin-Bs induces reverse signal transduction to presynaptic EphBs and promotes synapse formation (McClelland et al., 2009).

Because syntenin interacts with a variety of glutamate receptors (Hirbec et al., 2002, 2003; Enz and Croci, 2003), it is plausible that syntenin regulates synaptic function through the glutamate permeability of the postsynaptic membrane. Although the precise function of syntenin in regulating the activity and location of glutamate receptors is still to be elucidated, the identified syntenin functions support the idea that syntenin can target the interacting partners to subcellular locations, such as synapses, and regulate synaptic integrity by changing membrane architecture. A recent study showed the possible mechanism of syntenin-mediated glutamate receptor control. A cell adhesion protein, Coxsackievirus and adenovirus receptor (CAR), interacts with syntenin, and this interaction may induce the compartmentalization of the synapse by restricting the mobility of postsynaptic proteins such as glutamate receptors (Wrackmeyer et al., 2019). The increase in syntenin may cause an imbalance in CAR/syntenin interactions, resulting in changes in postsynaptic compartment and the permeability of glutamate to postsynaptic membranes.

## 5 Behavioral effects caused by mTORC1 or syntenin activation in TSC model animals

The representative neuropsychiatric disorders in TSC are refractory epilepsy, ASD, and intellectual disability. In addition, various symptoms are observed in TSC patients, including anxiety, depression, hyperactivity, aggression, sleep problems, etc., which are called “TSC-associated neuropsychiatric disorders (TANDs)” with meaningful criteria (Curatolo et al., 2015; Vanclooster et al., 2022). The precise mechanisms underlying the pathogenesis of these disorders are still elusive, but animal model studies have focused on the signaling cascade causing behavioral abnormalities that mimic TANDs and investigate possible pharmacological targets to improve symptoms. Heterozygous knockout of *Tsc1* or *Tsc2* (*Tsc1*^±^ or *Tsc2*^±^) in mice induced cognitive deficits and impaired memory, as shown by the results of the Morris water maze test and contextual fear discrimination test (Goorden et al., 2007; Ehninger et al., 2008). These learning deficits may well mimic the intellectual disability observed in many patients with TSC. For a model study of ASD, social behaviors were investigated in *Tsc1*^±^ and *Tsc2*^±^ mice, and mutant mice showed less social interaction with the other mice (Goorden et al., 2007; Sato et al., 2012). In addition, transgenic mice expressing the dominant-negative form of Tsc2 also showed less interaction time with the other mice (Chévere-Torres et al., 2012), indicating that the loss of function of Tsc1/2 imitates ASD symptoms in TSC patients. Other TAND symptoms were observed in TSC model mice. Elevated plus maze test and open field test results indicated that Tsc2 dominant-negative mice showed high anxiety and depressive mood (Ehninger and Silva, 2011). Several studies have shown that rapamycin treatment reversed learning deficits (Ehninger et al., 2008; Sato et al., 2012), indicating that the mTORC1 activation induced by a loss of Tsc1/2 function can be involved in model mouse TANDs. Based on the results of cellular analysis, synaptic excitability alteration by mTORC1 activation could cause abnormal neural function, leading to abnormal behavior. Rapamycin treatment alleviated cognitive deficits in Alzheimer’s disease model mice (Lin et al., 2017; Wang et al., 2021) and in autophagy-hyperactivated mice (Liu et al., 2017; Wang et al., 2019). Therefore, it is suggested that rapamycin ameliorates symptoms in TSC mice by regulating synapse function than by regulating spine morphogenesis. However, recent studies indicated controversial effects of rapamycin on behavioral abnormalities observed in mice. In fragile-X syndrome model mice, mTORC1 activation was observed and rapamycin was expected to normalize the abnormal behavioral phenotype of the mice. Nevertheless, rapamycin treatment had negative effects on behaviors, increasing hyperactivity and anxiety-like mood and impairing social interactions, and did not lead to the recovery of cognitive defects (Saré et al., 2017). Therefore, further analysis will be required to understand the relationships between mTORC1 activation and behavioral abnormalities in mouse models. Controversial results were also reported in rapamycin treatment on TANDs in TSC patients. Clinical trial reports showed that everolimus, a derivative of rapamycin, did not improve cognitive functioning, autism, or neuropsychological deficits in children with TSC (Krueger et al., 2017; Overwater et al., 2019), indicating that the effects of rapamycin observed in mice model were not quite reproduced in TSC patients. Taken together, more elucidation of the mTORC1-mediated development of neuropsychiatric symptoms in TSC is needed, and the detailed downstream factors or precise molecular and cellular mechanisms remain to be revealed.

Pharmacological inhibitors of syntenin have recently been reported (Leblanc et al., 2020; Haugaard-Kedström et al., 2021; Pradhan et al., 2021), but they are not widely used for functional analyses of syntenin. Therefore, knockdown or knockout animals have been used to analyze the effects of syntenin on learning deficits. The knockdown of *syntenin* through intracerebroventricular injection of siRNA against *syntenin* recovered learning deficits in the contextual fear discrimination test in *Tsc2*^±^ rats (Eker rats) (Shimada et al., 2019), whereas control scrambled siRNA injection did not ameliorate impaired memory. Interestingly, the knockdown of *syntenin* in WT rats induced learning disability. This finding is consistent with previous findings that the balance of the protein levels of syntenin and CASK is important in spine morphogenesis and synapse formation. In another study, *syntenin* knockout mice failed to exhibit fear extinction in the cued fear conditioning test and showed abnormal c-Fos expression in amygdalae after the fear experience (Talukdar et al., 2018). These results are consistent with previous findings that an adequate decrease in syntenin protein can reverse the effect of Tsc1/2 loss on spine morphogenesis and synapse formation and that an excess decrease in syntenin induces spine dysmorphogenesis and synaptic abnormalities. After many repetitive studies are carried out on pharmacological syntenin inhibition, the effects of pharmacological syntenin regulation on TSC neurons and TSC animals should be carefully examined in terms of behavioral and histological abnormalities.

Epilepsy is one of the major neuropsychiatric disorders of TSC. Originally, heterozygous *Tsc1* or *Tsc2* knockout (*Tsc1*^±^ or *Tsc2*^±^) mice showed no seizure episodes (Onda et al., 1999; Kobayashi et al., 2001), and only one report showed that approximately half of *Tsc1*^±^ mice showed spontaneously transient characteristics of epilepsy (Gataullina et al., 2016). On the other hand, in various cell-specific models, such as Nestin-Cre-mediated neural progenitors, Dlx5/6-Cre-mediated GABAergic neurons, and Emx1-Cre-mediated embryonic neuroepithelial cells, *Tsc1* homozygous knockout induced severe and chronic seizures in mice (Goto et al., 2011; Magri et al., 2011; Fu et al., 2012), and rapamycin treatment extended the survival of the mice and abolished epileptic episodes (Goto et al., 2011; Magri et al., 2011). Moreover, inducible CamkII-Cre/ERT2-mediated excitatory neuron-specific *Tsc1* knockout in adult mice resulted in seizures, and rapamycin acted as an antiepileptic drug (Koene et al., 2019). In addition, the results of clinical trials using everolimus support the idea that rapamycin-mediated mTORC1 inhibition could be effective for the treatment of refractory epilepsy in TSC patients (Saffari et al., 2019; Stockinger et al., 2021). In conclusion, mTORC1 signaling may well be involved in a mechanism inducing epilepsy, whereas syntenin-mediated signaling may be involved in a mechanism for ASD and intellectual disability. Further analysis will reveal the detailed molecular signaling involved in these neuropsychiatric disorders.

## 6 Effect of glia-neuron crosstalk

The different types of glial cells support the function of neurons through their individual roles. Astrocytes provide nutrition to neurons; supply neurons with glutamine, the precursor required for glutamate synthesis; and regulate the homeostasis of Na^+^, K^+^, etc. (Felix et al., 2020; Kruyer et al., 2022). Microglia participate in synaptic pruning (Paolicelli et al., 2011; Zhan et al., 2014) and synapse formation (Parkhurst et al., 2013) in brain development and regulate synapse dynamics during postnatal stages (Weinhard et al., 2018). Oligodendrocytes accelerate the neural transduction rate and may regulate dendritic spine number and dynamics (Zemmar et al., 2018). In TSC patients, subependymal nodules (SENs) and subependymal giant-cell astrocytomas (SEGAs) are observed as histopathological abnormalities (Kingswood et al., 2017; Hasbani and Crino, 2018). SEGAs contain variable amounts of glial fibrillary acidic protein (GFAP) (Lopes et al., 1996) and GFAP-positive cells (Phi et al., 2008), so-called giant cells. Cortical tubers are also neuropathological hallmarks of TSC patients. Cortical tubers include increased expression of GFAP, vimentin, and S100β, marker proteins of astrocyte, as well as higher numbers of astrocytes. The astrocytes in cortical tubers often present reactive phenotypes compared to the perituberal area and control brain tissue (Richardson, 1991; Talos et al., 2008). Therefore, it is likely that the glial cells harboring *Tsc1/2* mutations aggravate synaptic dysfunction and impede normal dendritic spine formation.

There has been no evidence that astrocyte-specific *Tsc1/2* mutations directly regulate synaptic function. However, a variety of studies using GFAP-Cre-mediated astrocyte-specific *Tsc1* or *Tsc2* knockout mice have indicated that the loss of function of Tsc1/2 indirectly regulates synaptic function through the regulation of the extracellular concentration of glutamate and potassium (Wong et al., 2003; Jansen et al., 2005; Zeng et al., 2007). Extracellular glutamate and potassium control the firing potentiation of postsynaptic neurons. Interestingly, GFAP-Cre-mediated astrocyte-specific *Tsc1* homogenous knockout mice exhibited epileptic seizures (Uhlmann et al., 2002; Wong et al., 2003), suggesting that the dysregulation of glutamate and potassium transporters in *Tsc1* knockout astrocytes increased the excitability of the neurons and decreased the seizure threshold to induce epilepsy. In addition, behavioral analysis showed that astrocyte-specific *Tsc1* knockout mice exhibited cognitive deficits in spatial learning (Zeng et al., 2007). Taken together, these results show that Tsc1/2 function in astrocytes to control the neuronal excitability by regulating the environment of synapses. Neuronal excitability alteration results in the induction of epilepsy and cognitive deficits. These inferences further suggest that impaired astrocyte function is well involved in neuropsychiatric symptoms in TSC patients.

Pathological studies have shown that active microglia are observed within tubers from TSC patients (Boer et al., 2008a,b). Active microglia are one of the hallmarks of brain inflammation. Thus, microglial activation may be a secondary effect of epileptic seizures. However, it is difficult to determine whether microglial activation is a primary pathophysiological mechanism inducing epilepsy or is simply a secondary effect to seizures. Recent studies using CX3CR1-Cre-mediated microglia-specific *Tsc1* knockout mice investigated the function of Tsc1/2 in microglia. Knockout mice demonstrated evidence of microglial activation, observed as increased cell size and an increased number of microglia in Iba1 immunohistochemical analyses (Zhang et al., 2018; Zhao et al., 2018). The mice had severe epilepsy and decreased survival. All the mice died by 13 weeks in one report (Zhang et al., 2018) and by 5 weeks in another report (Zhao et al., 2018). Rapamycin treatment almost completely suppressed the development of seizures and prolonged the survival of the mice, but once rapamycin administration was stopped, seizures subsequently recurred, and all mice died (Zhang et al., 2018). These results suggest that Tsc1/2 loss of function in microglia regulates neuronal excitability through microglial mTORC1 activation. In addition, the number of excitatory and inhibitory synapse marker spots in the hippocampus was decreased in knockout mice, indicating that synapse formation was affected by the function of *Tsc1/2*-mutated microglia (Zhao et al., 2018). Although a neurocognitive or behavioral phenotype has not been reported, the epileptic phenotype supports that Tsc1/2 in microglia can regulate synapse functions.

White matter abnormalities have emerged as an important and distinctive mechanism for brain dysfunction in TSC patients, indicating that the loss of function of Tsc1/2 in oligodendrocytes is involved in the histological pathogenesis of TSC. Hypomyelination and a decrease in oligodendrocyte number were observed in and around cortical tuber specimens (Scholl et al., 2017). In addition, animal studies using Olig2-Cre-mediated oligodendrocyte-specific *Tsc2* knockout mice showed hypomyelination and decreased oligodendrocyte numbers in the cortex and white matter tract (Carson et al., 2015). Conditional knockout mice did not exhibit an anxiety phenotype or communication deficits, but cognitive deficits were not investigated (Carson et al., 2015). Several studies on schizophrenia, brain injuries, and hypoxia have suggested that hypomyelination may be involved in learning disability (Xiao et al., 2016; Min et al., 2017; Maas et al., 2021). It is still unknown whether the loss of function of Tsc1/2 in oligodendrocytes is involved in synaptic regulation and spine morphogenesis.

Overall, the glial dysfunction induced by Tsc1/2 loss led mainly to synaptic abnormalities, especially increased excitability. Glial cells have important roles in neuropsychiatric symptoms in TSC. However, the precise mechanism regulating synapse function has not been fully revealed, and the effect of Tsc1/2-mutated glia on spine morphogenesis is not known. Further analyses are required to understand the function of Tsc1/2 in glial cells to regulate neuronal function, synapse control and spine morphogenesis.

## 7 Conclusion and future direction

The various studies on TSC have revealed precise molecular mechanisms of Tsc1 and Tsc2 proteins. Mainly signaling pathways regulating Tsc1/2 and those regulated by Tsc1/2 were well clarified. Additionally, the cellular mechanisms of benign tumor formation have been well elucidated. Mutations in *Tsc1* and *Tsc2* resulted in a loss of function of the Tsc1/2 complex and the activation of Rheb1, enhancing the activity of the mTORC1 complex. mTORC1 activity highly promoted cell proliferation and affected synapse function and neural excitability. Rapamycin normalized these abnormalities by inhibiting mTORC1. Therefore, the rapamycin derivative everolimus has been used as a therapeutic drug for SEGA formation, renal angiomyolipoma, and refractory seizures (Ruiz-Falcó Rojas et al., 2022).

The cellular mechanisms related to neuropsychiatric disorders and other TANDs in TSC patients are not well understood and remain to be elucidated. Rheb1-mediated increased syntenin level altered spine morphogenesis and synapse formation. Behavioral tests using *syntenin* knockout mice indicated that an excess increase in syntenin protein may lead to intellectual disability in animals. Therefore, syntenin is a promising therapeutic target for treating TSC symptoms. For a better understanding of syntenin in the neural function of TSC, further analysis is needed to determine whether increased syntenin is involved in inducing other TANDs in model animals, and thereafter, pharmacological inhibition of syntenin could normalize the cellular and behavioral defects of the animals.

Recently, a new perspective was proposed to regulate Rheb1 activity for the pharmacological treatment of TSC. Because Rheb1 is modified with farnesyl residues during activation and farnesylation is a necessary step for Rheb1 activity, inhibiting farnesylation by farnesyl transferase inhibitors (FTIs) could suppress active Rheb1-mediated neuropsychiatric disorders in TSC. The administration of lonafarnib, one of the FTIs, normalized spine dysmorphogenesis and abnormal synapse formation in *Tsc2*^±^ neurons and ameliorated learning deficits in *Tsc2*^±^ mice (Sugiura et al., 2022). Because Rheb1 signaling governs both the mTORC1 and syntenin signaling pathways, direct regulation of Rheb1 activity will be a way to develop therapeutic methods based on approaches other than the regulation of mTORC1 and syntenin. The combination of these therapeutic approaches will help clinical studies establish treatment plans.

In addition, research on glia-neural crosstalk in TSCs has just begun. Astrocyte-specific *Tsc1* knockout mice showed severe seizures, but the phenotype of the other glia-specific *Tsc1/2* knockout mice has rarely been investigated. To elucidate how glia-neuron crosstalk is involved in TSC symptoms, it is necessary to analyze the effect of *Tsc1/2* gene-deleted glia on neurons, especially spine formation, synapse function, and neural excitability. Furthermore, *in vivo* analysis should be carried out to clarify how glial defects caused by *Tsc1/2* mutation induce behavioral defects in model mice.

One of the large mysteries of the relationship between TSC model animals and TSC patients is the use of different gene dosages to promote pathogenesis. It is believed that heterozygous mutation of *Tsc1* or *Tsc2* results in the onset of TSC in humans; however, heterozygous knockout of *Tsc1* or *Tsc2* did not cause hamartoma formation or epilepsy in a mouse model. In *Tsc2*^±^ mice, spine dysmorphogenesis and synapse abnormalities were observed, and the mice showed cognitive deficits (Ehninger et al., 2008; Sugiura et al., 2015). Although spine morphology is not reported in postmortem analyses of TSC patients, spine dysmorphology is one of the hallmarks of neuropsychiatric symptoms. To explain the difference, one possibility is that the mechanisms connecting cellular abnormalities and pathological conditions may be different between mice and humans. Another possibility is the so-called “Knudson’s two-hit hypothesis” (Knudson, 1971). Spontaneous somatic mutation results in a loss of heterozygosity (homozygous knockout) and promotes tumor formation. Renal carcinomas can be induced by chemical mutation in *Tsc2*^±^ rats (Kobayashi et al., 1997). Hopefully, studies using mouse and human iPSC-derived organoids will help to clarify the detailed difference between mice and humans for a better understanding of TSC pathogenesis.

Another mystery is that the efficacy of rapamycin in humans is different from that in mice. Most pathogenic behavioral phenotypes observed in *Tsc1/2* mutant mice were normalized by rapamycin administration, but the effect of rapamycin on TSC patients was limited. Everolimus is still applied in the treatment of only a few symptoms of TSC, even benign tumor formations. Although it has often occurred that therapeutic drugs that are effective in animal models of disease fail to achieve the goals of clinical trials, the gene dosage anomaly may be one of the reasons for rapamycin ineffectiveness in human TSC patients. To establish better therapeutic methods, further analysis, such as detailed molecular signaling covering neuronal dysfunctions, the mechanisms connecting cellular and histological abnormalities to behavioral defects, and intercellular communications related to physiological pathogenesis, are needed.

Many studies have already found a wide variety of molecular functions of Tsc1/2; however, these findings are just the beginning in understanding the pathogenesis of the neuropsychiatric symptoms of TSC. Future elucidation of the histological and physiological function of Tsc1/2 will help to establish better pharmacological therapy for TSC patients.

## Author contributions

TS and KY developed the concept and designed the initial manuscript. TS wrote the manuscript and produced the figures. KY supervised the manuscript writing and editing. Both authors contributed to the article and approved the submitted version.
